# Pediatric Farm Injuries: Morbidity and Mortality

**Published:** 2017-11-30

**Authors:** Clint Rathje, Ashley Venegas, Stephen D. Helmer, Rachel M. Drake, Jeanette G. Ward, James M. Haan

**Affiliations:** 1 University of Kansas School of Medicine-Wichita Department of Surgery; 2 Via Christi Health, Wichita, KS; 3 Chandler Regional Medical Center, Chandler, Arizona

**Keywords:** farm, pediatrics, children, injury

## Abstract

**Background:**

Agriculture is an industry where family members often live and work on the same premises. This study evaluated injury patterns and outcomes in children from farm-related accidents.

**Methods:**

A 10-year retrospective review of farm-accident related injuries was conducted of patients 17 years and younger. Data collected included demographics, injury mechanism, accident details, injury severity and patterns, treatments required, hospitalization details, and discharge disposition.

**Results:**

Sixty-five patients were included; 58.5% were male and the mean age was 9.7 years. Median Injury Severity Score and Glasgow Coma Scale were 5 and 15, respectively. Accident mechanisms included animal-related (43.1%), fall (21.5%), and motor vehicle (21.5%). Soft tissue injuries, concussions and upper extremity fractures were the most common injuries observed (58.5%, 29.2%, and 26.2%, respectively). Twenty-six patients (40%) required surgical intervention. Mean hospital length of stay was 3.4 ± 4.7 days. The majority of patients were discharged to home (n = 62, 95.4%) and two patients suffered permanent disability.

**Conclusions:**

Overall, outcomes for this population were favorable, but additional measures to increase safety, such as fall prevention, animal handling, and driver safety training should be advocated.

## Introduction

Agriculture is one of the few industries where family members often live and work on the same premises. In 2012, there were approximately 2.2 million farms in the United States.^1^ An estimated 955,000 youth under 20 lived on a farm and 49% performed farm work.^2^ Another estimated 259,000 nonfarm resident youth were hired to work on United States farms in 2012. This was a 12.4% increase from the 230,400 hired youth in 2009.^1^,^2^ Due to living and working in close proximity to animals, chemicals and dangerous machinery, this puts them at increased risk for serious injury, disability, and even death.

In the United States, 45 children are injured every day and another dies every three days in an agricultural-related incident.^3^ The fatal work-related injury rate for youth in agriculture is 3.6 times higher than that of all other industries, and 2.9 times higher than adult workers in all other industries combined. The Midwest region is estimated to have a higher proportion of youth on farms than any other region and, as such, is found to have the highest number of pediatric farm-related injuries in the United States.^4^ Kansas is mostly a rural state and ranks seventh in agricultural production in the United States.

To date, efforts to describe the risk for injury to youth living and working on farms have largely come from Canadian populations^5^–^9^, where a national database of farm injuries is available. Findings from these studies suggest the most common mechanisms for farm injuries are dependent on several factors. Chief among these are age of the child, developmental level, and the immediate environment (work setting vs non-work setting).^6^, ^10^–^13^ These factors interact with one another to create distinct injury profiles. Prevention measures based on these profiles can provide a targeted way to reduce preventable agricultural injuries; however, the research on effective interventions is still in its infancy.^14^ Furthermore, the generalizability of studies based largely on Canadian agricultural practices to the Midwestern United States is uncertain. Therefore, the purpose of this study was to describe and compare farm injuries in Kansas with international data to determine consistency and provide specific trauma information on farm-related injuries and outcomes which will be useful in the development of injury prevention measures.

## Methods

A retrospective review was conducted of all pediatric patients (<18 years of age) who presented with farm-accident related injuries at an American College of Surgeons verified level 1 trauma center between January 1, 2004 and December 31, 2013. Patients were identified and data were retrieved from the trauma registry, as well as from patient medical records. Patient data included age, gender, race, location where injury occurred (ICD-9-CM code E849.1, Farm), type of injury (blunt vs. penetrating), mechanism of injury, initial Glasgow Coma Scale (GCS) score, initial Injury Severity Score (ISS), initial vital signs (blood pressure, respiration, pulse, oxygen saturation), blood product type and total in-hospital units, alcohol and drug screen results, mode of transportation (EMS ground, fixed wing airplane, helicopter ambulance), injury details, intensive care unit (ICU) admission and length of stay, ventilatory requirements (i.e., need for ventilation and duration), need for and type of operative or procedural management, complications, hospital length of stay, disabilities, discharge disposition (i.e., home, rehabilitation, or acute care hospital), and mortality.

This study was approved for implementation by the Institutional Review Board of Via Christi Hospitals Wichita, Inc. Data from patient medical records were abstracted and summarized. Continuous data are reported as the mean ± the standard deviation or the median with interquartile range, when data are skewed. Categorical data are presented as raw counts with percentages noted parenthetically. All descriptive analyses were conducted using SPSS release 19.0 (IBM Corp, Somers, New York).

## Results

Sixty-five patients were identified as pediatric farm traumas. The majority was male (58.5%) and suffered blunt trauma (93.8%); the average age was 9.7 years (Table 1). Twenty-nine (44.6%) were aged 0 – 9 years and 36 (55.4%) were between the ages of 10 and 17 years. The majority of these patients were brought to the hospital via private vehicle. Many of these traumas were animal-related (43.1%), which mainly involved riding an animal such as a horse. One patient tested positive for alcohol and another for drugs. In general, patients suffered minor injuries as evidenced by the median ISS of 5.

Concussion and loss of consciousness were relatively common (29.2% and 24.6%, respectively) while traumatic brain injury was a rare event, only occurring in two patients (Table 2). Of these patients, one was found to have a subarachnoid hemorrhage, while the second suffered multiple small parenchymal hemorrhages and a subarachnoid hemorrhage. The majority of these injuries were the result of falls and animal or motor-vehicle accidents.

The majority of injuries were musculoskeletal in nature involving soft tissue injuries (n = 38) and fractures (n = 36; Table 2). Of the soft tissue injuries, most involved abrasions or lacerations that required suturing. The most common mechanism for this injury was animal-related. One of the more significant injuries involved a degloving of a left upper extremity secondary to a motor-vehicle collision. This person ultimately required a skin graft.

As for fractures, the upper extremity (n = 17) was involved more commonly and was mostly the result of animal-related incidents (Table 2). Other fractures included lower extremity (n = 7), pelvis (n = 4), spine (n = 4), and ribs (n = 4). These orthopedic injuries accounted for the majority of surgical interventions (n = 19, 73%). Some of the more serious orthopedic injuries involved spinal fractures. Two of these patients’ injuries were due to falls and required surgical intervention on the cervical and thoracic spine, respectively. Unfortunately, the patient that required surgical stabilization of the thoracic spine suffered permanent disability. Another patient with a permanent disability involved an animal-related incident that resulted in a traumatic brain injury.

Intraabdominal injuries were uncommon (Table 2). There were four splenic injuries involving motor vehicle or animal-related trauma. Only one of these patients required intervention with embolization. Liver injuries were found in two animal-related incidents and were managed conservatively. One patient with a hollow viscus injury secondary to a gunshot wound required surgical intervention with exploratory laparotomy. This person received one unit of blood.

Just over one-third (n = 23; 35.4%) of patients were admitted to the ICU and nine (13.8%) required mechanical ventilator support (Table 3). The average hospital length of stay was 3.4 ± 4.7 days. The majority of these patients (n = 63; 96.9%) were discharged home after their hospitalization, although two suffered permanent disability from their injuries. There were no deaths.

## Discussion

Agricultural injuries are difficult to research as there is no central database tracking these types of injuries. Therefore, most of the data is obtained from a Childhood Agricultural Injury Survey (CAIS) that is organized through the collaboration of the National Institute for Occupational Safety and Health (NIOSH), the U.S. Department of Agriculture, and the National Agricultural Statistics Service (USDA-NASS), or international studies.^1^,^4^,^5^,^7^,^9^,^13^ The objective for this study was to describe the injury profiles associated with pediatric farm injuries in the state of Kansas and compare those profiles to previous literature and international studies.

The most common injuries in our population were soft tissue injuries, fractures, and concussions. Concussions were common head injuries in preadolescent farm residents/workers, with younger children (< 6 years of age) suffering the most severe head injuries. 9,15 Upper extremity fractures were the most common type of fracture in our study, which confirmed the findings from a study of fractures and amputations caused by farm equipment.^16^ The leading causes of nonfatal injuries were related to falls, animals, and machinery. Horses and all-terrain vehicles were major contributors to the latter two categories. This is comparable to the extant literature.^2^,^8^,^9^,^17^

Earlier studies found that these injuries may stratify differently depending on age and gender.^6^,^9^ Historically, males are injured more commonly, and this was true with our study as well; however, the leading cause of preadolescent (> 10 years) injury in females was animal related whereas machinery was the leading cause in preadolescent males.^9^,^15^,^16^ Injuries from falls or jumps were most common in those under the age of 10 years. Authors have explained that the differences in injury patterns amongst these groups likely is related to how age and gender differences dictate children’s environmental or work hazard exposures.^9^,^11^,^13^ Children under the age of 10 are less likely to be in the immediate work environment (e.g., working with animals, machinery), but can be injured while playing on structures not intended for entertainment (e.g., a hayloft).

Long-term disability was found in 3.1% of patients compared to the national data of approximately 5%.^2^,^3^ The injuries associated with long-term disability typically included traumatic brain injury, spinal cord injury, limb amputation, or crush injury.^2^,^3^ No patients died in this study. However, NIOSH reported that an average of over 100 youth die annually from farm injuries.^2^,^3^ Two major sources of fatality were crush injuries to the head, abdomen, or chest from machinery and motor vehicles, including all-terrain vehicles. The most common vehicular related fatalities involved tractors, which accounted for one-third of all deaths.^3^ The third most common contributor to agricultural-related deaths was drowning.^1^,^17^

Farm children are put in a unique position as they live, play, and work in an environment that is surrounded by animals, chemicals, and dangerous machinery. This unique environment places them at increased risk for serious injury or disability. When children are not socially, cognitively, or physically developed enough to navigate this work environment, the potential for injury can increase dramatically.^13^ Even if a child is not within or near the immediate work area, the necessity for supervision and monitoring can create less than ideal safety conditions for children and/or their parents.^12^ A number of research lines have focused on injury prevention strategies. While the work is promising, authors highlighted the need for more comprehensive databases that can be used for the development and evaluation of targeted interventions. For example, NIOSH developed a comprehensive childhood agricultural injury prevention initiative.^18^ A key component of this initiative was the development of “infrastructure that facilitates the use of data and research results to develop and improve prevention efforts”.^18^ This and other efforts to improve the safety of agriculture are imperative to the safety of our youth, especially in the areas of driver safety, animal handling and fall prevention.

While our study takes a step toward meeting the larger national objective set forth by NIOSH, it is not without limitations. First, our findings were based on a limited data set from one institution, and may not illustrate the range and frequency of farm injuries fully from all of Kansas. Secondly, our small sample size limited our ability to draw any statistical inferences beyond simple description of the frequency and type of injury. Lastly, the retrospective nature of the study design limited our ability to gather pertinent information regarding factors and/or situational circumstances that may be associated with the injuries, such as whether the youth were injured while engaged in farming activities or injured while on the farm, but not engaged in farm-related work activities. This limits, to some extent, what can be inferred about the causal events of youth farm injuries; however, earlier works on injury prevention can provide a template on how to develop appropriate interventions. Therefore, future work in this area should focus on prospective studies using data from multiple sources serving rural Kansas.

## Conclusions

Youth that live and/or work in agricultural settings are at an increased risk for serious injury, especially falls, injury from animals, and injuries from dangerous machinery. Continued efforts to develop and evaluate targeted injury prevention strategies should be a focus of health researchers in Kansas.

## Figures and Tables

**Table 1:** Study patient demographics, injury severity and mechanism, and initial vitals.

Number of patients [n (%)]	65 (100.0%)

Age, years [Mean ± SD]	9.7 ± 4.8

Male sex [n (%)]	38 (58.5%)

Race (Caucasian) [n (%)]	61 (93.8%)

Mode of transportation [n (%)]	
Private vehicle	37 (56.9%)
Ground ambulance	19 (29.2%)
Helicopter	9 (13.8%)

Injury Severity Score (ISS) [Median (25^th^ and 75^th^ percentiles)]	5 (4, 10)

Initial Glasgow Coma Scale (GCS) Score [Median (25^th^ and 75^th^ percentiles)]	15 (15, 15)

Mechanism (blunt/penetrating) [n (%)]	61 (93.8%)/4 (6.2%)

Type of accident [n (%)]	
Animal-related	28 (43.1%)
Fall	14 (21.5%)
Motor-vehicle accident	14 (21.5%)
Struck	4 (6.2%)
Gunshot wound	3 (4.6%)
Machine	1 (1.5%)
Cut	1 (1.5%)

Admission Vitals [Mean ± SD]	
Systolic blood pressure (mmHg)	122.7 ± 19.9
Diastolic blood pressure (mmHg)	78.2 ± 17.0
Respiratory rate (breaths per minute)	20.7 ± 7.7
Heart rate (beats per minute)	105.4 ± 25.0
Oxygen saturation (%)	98.6 ± 1.6

**Table 2:** Farm injury characteristics of study patients (n = 65).

Injury Parameter [n (%)]	
Traumatic brain injury	2 (3.1%)
Concussion	19 (29.2%)
Loss of consciousness	16 (24.6%)
Neurologic deficit	6 (9.2%)
Spine fracture	4 (6.2%)
Spinal cord injury	2 (3.1%)

Thoracic injuries [n (%)]	
Cardiac injury	1 (1.5%)
Pulmonary contusion	4 (6.2%)
Pneumothorax	7 (10.8%)
Hemothorax	3 (4.6%)
Rib fracture	4 (6.2%)
Bilateral rib fracture	0 (0.0%)

Abdominal injuries [n (%)]	
Spleen	4 (6.2%)
Liver	2 (3.1%)
Hollow viscus	1 (1.5%)
Pancreatic/biliary	0 (0.0%)
Renal	0 (0.0%)
Other genitourinary	2 (3.1%)

Pelvic fracture [n (%)]	4 (6.2%)

Upper Extremity Fractures or Dislocations [n (%)]	17 (26.2%)

Lower Extremity Fractures or Dislocations [n (%)]	7 (10.8%)

Soft tissue injury [n (%)]	38 (58.5%)

**Table 3:** Characterization of Hospitalization Details and Disposition (n = 65).

Hospital parameter	
Intensive care unit (ICU) admission [n (% )]	23 (35.4%)
ICU length of stay, in days (all patients, n = 65) [Mean ± SD]	1.2 ± 2.6
Mechanical ventilation [n (% )]	9 (13.8%)
Mechanical ventilation days (all patients, n = 65) [Mean ± SD]	0.4 ± 1.8
Surgery [n (% )]	40.0% (26)
Procedures [n (% )]	30.8% (20)
Blood transfusion [n (% )]	1.5% (1)
Complication [n (% )]	4.6% (3)
Permanent disability [n (% )]	3.1% (2)
Hospital length of stay, in days (all patients, n = 65) [Mean ± SD]	3.4 ± 4.7

Disposition	
Home	62 (95.4%)
Rehabilitation	2 (3.1%)
Home with home health	1 (1.5%)
Death	0 (0.0%)

## References

[1] National Children’s Center for Rural and Agricultural Health and Safety (2013). Childhood agricultural injuries in the U.S., 2014 Fact Sheet.

[2] Centers for Disease Control and Prevention, National Institute for Occupational Safety and Health Agricultural safety.

[3] Wright S, Marlenga B, Lee BC (2013). Childhood agricultural injuries: An update for clinicians. Curr Probl Pediatr Adolesc Health Care.

[4] Centers for Disease Control and Prevention, National Institute for Occupational Safety and Health (2013). Childhood agricultural injury survey (CAIS) results. Childhood agricultural injury prevention initiative.

[5] Marlenga B, Brison RJ, Berg RL, Zentner J, Linneman J, Pickett W (2004). Evaluation of the North American Guidelines for Children’s Agricultural Tasks using a Case Series of Injuries. Inj Prev.

[6] Morrongiello BA, Marlenga B, Berg R, Linneman J, Pickett W (2007). A new approach to understanding pediatric farm injuries. Soc Sci Med.

[7] Pickett W, Brison RJ, Berg RL, Zentner J, Linneman J, Marlenga B (2005). Pediatric farm injuries involving non-working children injured by a farm work hazard: Five priorities for primary prevention. Inj Prev.

[8] Pickett W, Dostaler S, Berg RL, Linneman JG, Brison RJ, Marlenga B (2007). Pediatric fall injuries in agricultural settings: A new look at a common injury control problem. J Occup Environ Med.

[9] Pickett W, Dostaler S, Berg RL, Brison RJ, Linneman JG, Marlenga B (2008). Hospitalized head injuries in agricultural settings: Who are the vulnerable groups?. Accid Anal Prev.

[10] Brison RJ, Pickett W, Berg RL, Linneman J, Zentner J, Marlenga B (2006). Fatal agricultural injuries in preschool children: Risks, injury patterns and strategies for prevention. CMAJ.

[11] Morrongiello BA, Zdzieborski D, Stewart J (2012). Supervision of children in agricultural settings: Implications for injury risk and prevention. J Agromedicine.

[12] Morrongiello BA, Pickett W, Berg RL, Linneman JG, Brison RJ, Marlenga B (2008). Adult supervision and pediatric injuries in the agricultural worksite. Accid Anal Prev.

[13] Schwebel DC, Pickett W (2012). The role of child and adolescent development in the occurrence of agricultural injuries: An illustration using tractor-related injuries. J Agromedicine.

[14] Hartling L, Brison RJ, Crumley ET, Klassen TP, Pickett W (2004). A systematic review of interventions to prevent childhood farm injuries. Pediatrics.

[15] Centers for Disease Control and Prevention, National Institute for Occupational Safety and Health (2014). Childhood agricultural injury survey (CAIS) results. CAIS injury tables. Childhood agricultural injury prevention initiative.

[16] Lubicky JP, Feinberg JR (2009). Fractures and amputations in children and adolescents requiring hospitalization after farm equipment injuries. J Pediatr Orthop.

[17] Smith GA, Scherzer DJ, Buckley JW, Haley KJ, Shields BJ (2004). Pediatric farm-related injuries: A Series of 96 Hospitalized Patients. Clin Pediatrics.

[18] Hard DL, Robison WA A Summary of NIOSH childhood agricultural injury prevention extramural research under the Childhood Agricultural Injury Prevention Initiative: A quindecennial (1997–2011) of progress.

